# Evaluation of the Efficacy and Cross-Protectivity of Recent Human and Swine Vaccines against the Pandemic (H1N1) 2009 Virus Infection

**DOI:** 10.1371/journal.pone.0008431

**Published:** 2009-12-23

**Authors:** Philippe Noriel Q. Pascua, Min-Suk Song, Jun Han Lee, Kuk Jin Park, Hyeok-il Kwon, Yun Hee Baek, Seung-Pyo Hong, Jong-Bok Rho, Chul-Joong Kim, Haryoung Poo, Thomas S. Ryoo, Moon-Hee Sung, Young Ki Choi

**Affiliations:** 1 College of Medicine and Medical Research Institute, Chungbuk National University, Cheongju, Republic of Korea; 2 Bioleaders Corporation, Daejeon, Republic of Korea; 3 College of Veterinary Medicine, Chungnam National University, DaeJeon, Republic of Korea; 4 Korean Research Institute of Bioscience and Biotechnology, Daejeon, Republic of Korea; 5 Prestige World Genetics, Genetics Korea, Ltd., Pyongtaek, Republic of Korea; Institut Pasteur Korea, Republic of Korea

## Abstract

The current pandemic (H1N1) 2009 virus remains transmissible among humans worldwide with cases of reverse zoonosis, providing opportunities to produce more pathogenic variants which could pose greater human health concerns. To investigate whether recent seasonal human or swine H1N1 vaccines could induce cross-reactive immune responses against infection with the pandemic (H1N1) 2009 virus, mice, ferrets or mini-pigs were administered with various regimens (once or twice) and antigen content (1.77, 3.5 or 7.5 µg HA) of a-Brsibane/59/07, a-CAN01/04 or RgCA/04/09xPR8 vaccine. Receipt of a-CAN01/04 (2-doses) but not a-Brisbane/59/07 induced detectable but modest (20–40 units) cross-reactive serum antibody against CA/04/09 by hemagglutinin inhibition (HI) assays in mice. Only double administration (7.5 µg HA) of both vaccine in ferrets could elicit cross-reactivity (30–60 HI titers). Similar antigen content of a-CAN01/04 in mini-pigs also caused a modest ∼30 HI titers (twice vaccinated). However, vaccine-induced antibody titers could not suppress active virus replication in the lungs (mice) or virus shedding (ferrets and pigs) of immunized hosts intranasally challenged with CA/04/09. Furthermore, neither ferrets nor swine could abrogate aerosol transmission of the virus into naïve contact animals. Altogether, these results suggest that neither recent human nor animal H1N1 vaccine could provide complete protectivity in all animal models. Thus, this study warrants the need for strain-specific vaccines that could yield the optimal protection desired for humans and/or animals.

## Introduction

Influenza A virus is the cause of recurrent influenza epidemics and from time to time, global pandemics. In the past century, the world had experienced three devastating influenza pandemics which claimed hundreds of thousands to millions of lives globally: Spanish Flu (H1N1, 1918–1919), Asian Flu (H2N2, 1957), and Hong Kong Flu (H3N2, 1968) [1]. A global pandemic was declared anew last June 11, 2009 by the World Health Organization (WHO) due to the emergence and rapid worldwide spread of a novel influenza A (H1N1) virus, hereafter referred to as pandemic (H1N1) 2009 virus [2], [3]. Although majority of laboratory-confirmed infections result in self-limiting, uncomplicated influenza [4], [5], others require hospitalizations or have fatal outcomes due to underlying medical conditions. Through animal models, experts provided evidence that the virus is pathogenic in mammalian hosts like mice, ferrets, and non-human primates [6]–[8] to extent even more higher than seasonal human influenza [6].

Detailed genomic sequence analysis of the pandemic (H1N1) 2009 virus reveals that it contains unique reassortment of genes that are of swine origin [9], [10]. Consequently, pigs (both commercial and specific-pathogen-free) are susceptible and can transmit the virus [6], [11], [12]. Since its identification in April 2009, reports of natural reverse zoonosis cases into pigs (Canada, Australia, United Kingdom, Ireland, Norway, Japan, Iceland, and most recently, the State of Indiana in the United States) and into breeding turkeys (Chile and Canada) have been considerably increasing [13]. Although the mortality rate due to infection with the pandemic virus among humans is low at present, establishment of the pandemic virus in a new host may yield more virulent strains. Pigs are strongly heralded as “mixing vessels” for the exchange of genetic materials between human and animal influenza viruses [14]–[17] potentially enhancing pathogenicity and lethality of the reassortant virus.

Vaccination is the primary measure to control influenza virus infections which come in two forms: inactivated or live-attenuated vaccine. Annually updated influenza virus vaccines typically contain three influenza viruses (trivalent): one A (H3N2) virus, one A (H1N1) and one B virus as chosen by the WHO Global Influenza Surveillance Network [18]. However, preliminary serological analyses suggest that contemporary seasonal influenza vaccines might not provide protective immunity to infection with the novel virus [10], [19], [20], [21]. Alternatively for human infections, antiviral agents are used as chemoprophylaxis for individuals who have not been vaccinated or for when a vaccine is not available. Although majority of isolated pandemic (H1N1) 2009 virus is responsive to neuraminidase inhibitors and are resistant to adamantanes, sporadic oseltamivir-resistant viruses are being isolated worldwide [22].

In the present study, we made use of mice, mini-pigs, and ferret animal models to assess the immunogenicity, protective efficacy, and cross-reactivity of various regimens of vaccination with inactivated whole-virus vaccines intended for humans (a-Brisbane/59/07) or for swine (a-CAN01/04). Results were compared to data obtained with RgCA/04/09xPR8 immunization, a reverse genetics-generated vaccine. Immunogenicity and cross-reactivity of the vaccines were evaluated by hemeagglutination assays (HI) while cross-protection in vaccinated animals were determined by challenge with the A/California/04/2009 virus. We report here and provide evidence of the inability of recent human and animal influenza A/H1N1 vaccines to provide complete protection, including inhibition of virus replication and transmission, among vaccinated mammalian hosts.

## Results

### Immunogenicity and Protection of a 2008–2010 Seasonal Human H1N1 Vaccine against the Pandemic (H1N1) 2009 Virus in Mice

**Figure 1:**
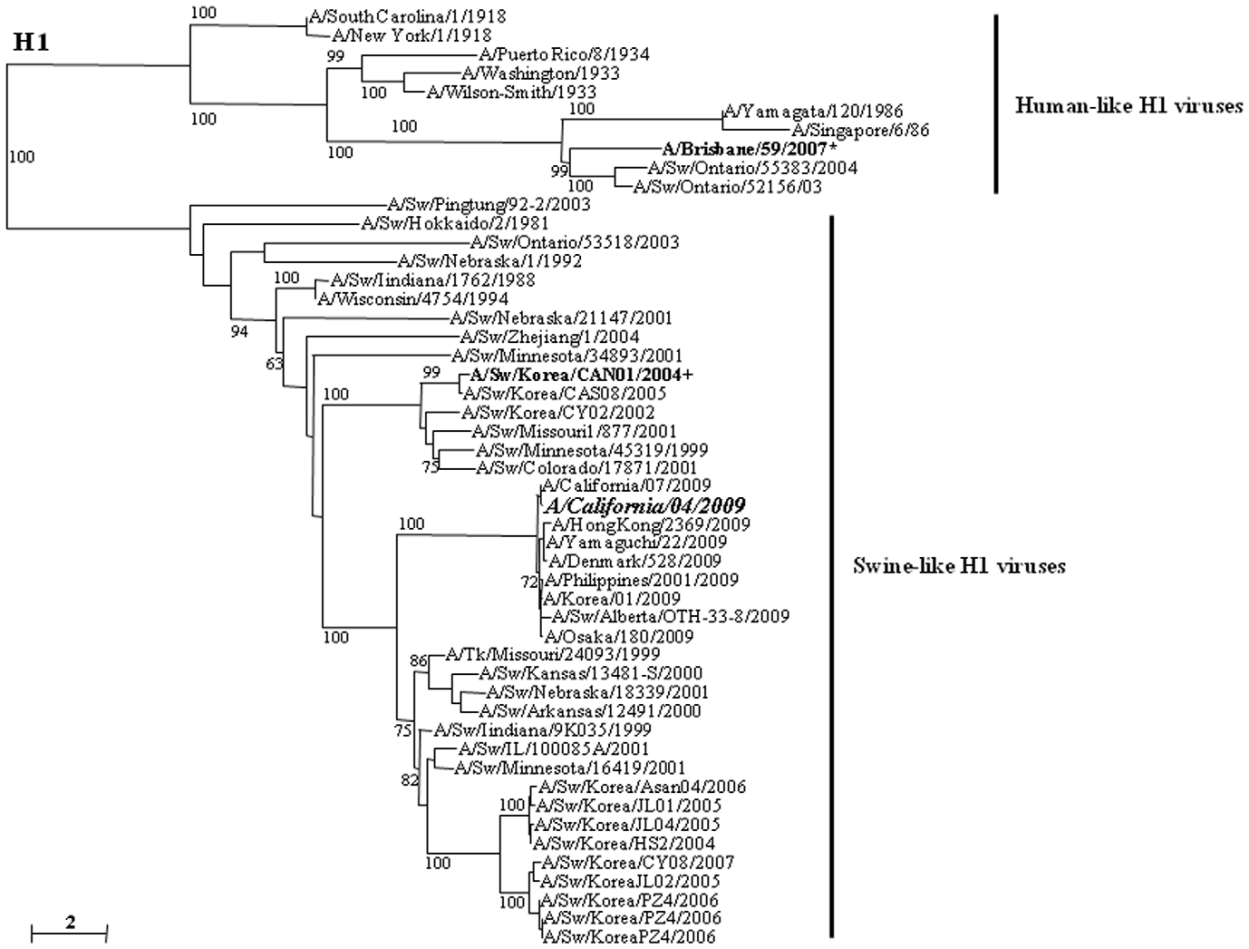
Alignment of the HA1 portion of the HA molecule of H1 influenza viruses. The amino acid sequences were aligned using Clustal_X [34], [35], and the phylogenetic tree was generated by the neighbor-joining method using the tree drawing program NJ plot [36]. The scale represents the number of substitutions per nucleotide. Branch labels record the stability of the branches over 100 bootstrap replicates. Only bootstrap values ≥60% was shown in each tree. The A/California/04/2009 virus shares about 70% and 88.4% amino acid sequence homology with the seasonal human (A/Brisbane/59/2007*) and Korean swine (A/Swine/Korea/CAN01/2004+) H1N1 viruses, respectively.

Serum specimens collected from children and adults that were immunized with recent seasonal influenza vaccines suggest that receipt of such vaccines is unlikely to elicit protective antibody immune response to the pandemic (H1N1) 2009 virus [19], [20]. To formally demonstrate this in our study, we determined the immunogenicity of a seasonal human H1N1 vaccine seed virus in the north (2008–2010) and south (2009) hemispheres in mice. Groups of 12 mice were vaccinated once or twice with 1.77 or 3.5 µg/dose HA of a-Brisbane/59/07 or RgCA/04/09xPR8, a reassortant vaccine virus generated by reverse genetics methods, containing 2% aluminum hydroxide adjuvant. By comparison, A/Brisbane/59/2007 (Brisbane/59/07) has a divergent genetic lineage to A/California/04/2009 (CA/04/09) with 70% amino acid identity in their HA H1 (Fig. 1). Two weeks after the last vaccination, mice sera were collected to determine the mean serum antibody response against Brisbane/59/07 or CA/04/09 by HI assays (Table 1). In general, mean HI titers appear to be enhanced in a dose-dependent manner. Results indicated that viruses homologous to the vaccine strains induced high mean HI titers particularly at two-dose administrations of 3.5 µg HA contents (∼320 HI units). However, none of the vaccine regimens of a-Brisbane/59/07 could elicit detectable cross-reactive immune response beyond the limit of detection (HI titer <20) against the CA/04/09 virus in mice sera. Vice versa, mice receiving a booster shot (double-dose groups) of RgCA/04/09xPR8 could only raise modest mean antibody titers (20-40 HI units) against the human seasonal H1N1 virus.

**Table 1:** Serum antibody response in mice after administration with inactivated vaccines.

Vaccine	Vaccine regimen	Mean serum antibody titer against			
	HA content	Brisbane/59/07	CAN01/04	CA/04/09	
a-Brisbane/59/07	Single dose				
	1.77 µg	<10	<10		<10
	3.5 µg	20–40	<10		<10
	Double doses				
	1.77 µg	80–160	40		<10
	3.5 µg	320	80		<10
a-CAN01/04	Single dose				
	1.77 µg	<10	<10		<10
	3.5 µg	<10	30–40		<10
	Double doses				
	1.77 µg	40	80–160		20
	3.5 µg	80	320		40
RgCA/04/09xPR8	Single dose				
	1.77 µg	<10	<10		<10
	3.5 µg	<10	<10		40
	Double doses				
	1.77 µg	20	20		160
	3.5 µg	40	40		320

Four-week-old BALB/c mice (12 heads per group) were immunized with inactivated whole-virus a-Brisbane/59/07, a-CAN01/04 and RgCA/04/09xPR8 vaccines containing 1.77 or 3.5 µg of HA administered once or twice with aluminum hydroxide adjuvant. Serum samples were collected 2 weeks after the last vaccination. The limit of detection for the HI assays done was set to <20 HI units and HI titers are expressed as the reciprocal of the highest dilution of serum that inhibits 8 HA units of virus (e.g., as 80 versus 1∶80).

To investigate the protective efficacy of the vaccine, immunized mice were intranasally (i.n.) challenged with 30ul of 10^5^ 50% tissue culture infective dose per milliliter (TCID_50_/ml) of the wild type CA/04/09 virus and monitored active virus replication in the lungs. A group of mock-vaccinated mice which only received phosphate-buffered saline (PBS) was included as control. Mice lungs were collected at 2, 4, 6, and 8 days post infection (dpi) (3 heads per day) for virus titration in 11-day-old embryonated chicken eggs. Experimentally challenged mice manifested clinical signs with influenza-like disease (i.e., inactivity, ruffled and erect hair) which was more prominent in mock-vaccinated and single-shot groups. Mock-vaccinated mice produced high lung titers initially detected at 2 dpi and persisting up to 8 dpi [7.0 and 4.3 log_10_ 50% egg infectious dose (EID_50_) virus titers, respectively]. Compared to a-Brisbane/59/07, RgCA/04/09xPR8 demonstrated the most efficient suppression of active virus growth in mice lungs in all of the vaccine regimens. Essentially, the pandemic (H1N1) 2009 virus was cleared from the lungs as early as 6 dpi in two-shot regimens with antigen containing as low as 1.77 µg/dose. Virus clearance was delayed in single shot groups of the reverse genetics vaccine (at 8 dpi) (Table 2). In contrast, replication of the challenge virus in mice vaccinated with a-Brisbane/59/07 could not completely inhibit virus replication regardless of vaccine regimen or antigen content allowing the CA/04/09 virus to persist up to 8 days. Lung titers were slightly reduced relative to the mock-vaccinated group (1–2 log_10_ EID_50_ lower). However, viral titers are still considerably higher compared to groups that received the RgCA/04/09xPR8. These results suggest that a-Brisbane/59/07 could not induce cross-reactive antibodies, sufficient to provide protection against infection or abrogate replication of the heterologous pandemic (H1N1) 2009 virus in mice lungs.

**Table 2:** Virus titers in the lungs of vaccinated mice.

Vaccine regimen and HA content	Days post infection			
	2	4	6	8
One-shot groups				
1.77 µg HA				
a-Brisbane/59/07	6.5 (0.3)*	5.7 (0.5)	4.5 (0.3)	3.2 (0.3)
a-CAN01/04	6.3 (0.3)	5.7 (0.5)	3.7 (0.3)	2.5 (0.5)
RgCA/04/09xPR8	5.0 (0.3)	4.1 (0.5)	2.1 (0.3)	0
3.5 µg HA				
a-Brisbane/59/07	5.7 (0.5)	4.3 (0.3)	3.7 (0.3)	2 (0.2)
a-CAN01/04	5.7 (0.5)	4.3 (0.3)	2.8 (0.5)	1.5 (0.3)
RgCA/04/09xPR8	3.5 (0.3)	2.7 (0.3)	0	0
Two-shot groups				
1.77 µg HA				
a-Brisbane/59/07	6.0 (0.3)	4.7 (0.3)	3.3 (0.5)	2.5 (0.5)
a-CAN01/04	6.0 (0.5)	4.3 (0.3)	2.7 (0.3)	1.8 (0.3
RgCA/04/09xPR8	3.7 (0.3)	2.1 (0.3)	0	0
3.5 µg HA				
a-Brisbane/59/07	5.3 (0.5)	3.7 (0.5)	2.7 (0.3)	1.5 (0.5)
a-CAN01/04	5.3 (0.3)	4.0 (0.5)	2.3 (0.3)	1 (0.5)
RgCA/04/09xPR8	2.3 (0.5)	1.2 (0.3)	0	0
Mock-vaccinated group	7.0 (0.5)	6.3 (0.5)	5.7 (0.3)	4.3 (0.3)

All vaccinated mice were intranasally (i.n.) challenged with 30 µl 10^5^ TCID_50_/ml of the CA/04/09 virus 2 weeks after the last immunization. Lung tissue samples were obtained at 2, 4, 6, and 8 days of virus challenge. Virus titers were measured in 11-day-old embryonated chicken eggs expressed as log_10_ EID_50_/g. The limit of virus detection was set to 0.7 log_10_ EID_50_/g.

* Standard deviation titers.

### Immunogenicity and Protection of the Seasonal Human H1N1 Vaccine in Ferrets

**Figure 2:**
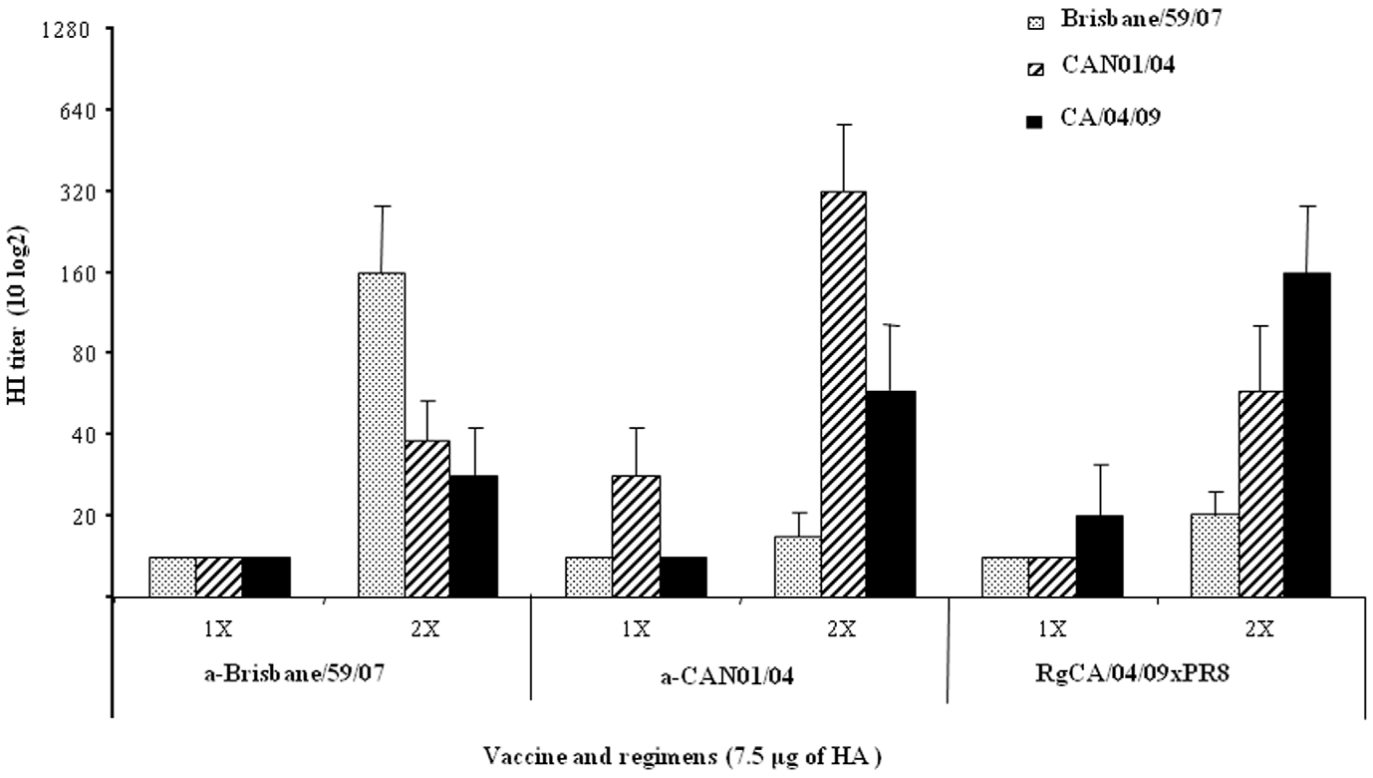
Hemagglutinin inhibition (HI) titers in ferrets administered with inactivated vaccines (a-Brisbane/59/07, a-CAN01/04, RgCA/04/09xPR8). Groups of four 15- to 16-week-old ferrets were vaccinated intramuscularly with one or two doses each of inactivated vaccines containing 7.5 µl/dose of HA with 2% of aluminum hydroxide adjuvant, administered 2 weeks apart. Sera were collected from recipients after 2 weeks the last vaccine was administered and mean hemagglutinin inhibition (HI) titers against Brisbane/59/07, CAN01/04 and CA/04/09 viruses were determined (limit of detection: <20 HI units) expressed as the reciprocal of the highest dilution of serum that inhibits 8 HA units of virus (e.g., as 80 versus 1∶80). Data are mean ± standard deviation titers.

Ferrets are broadly accepted as suitable animal models for influenza virus infection and transmission in humans [10], [23]. To re-affirm serologic analysis done in previous reports [19], [20], [21], groups of four 15- to 16-week-old ferrets were vaccinated once or twice with 7.5 µg/dose of HA with aluminum hyroxide adjuvant, administered 2 weeks apart intervals for two-dose. After 2 weeks of receiving the last vaccination, the mean HI titers against CA/04/09 and Brisbane/59/07 were determined in ferret sera (Fig. 2). Similar to the results obtained in mice, increased quantity and frequency of antigen administration raised antibody titers, more notably against the homologous H1N1 virus. In single dose recipients, both vaccines induced detectable but modest serum antibody titers in a homologous manner only (∼20 HI units) with no cross-reactivity. Accordingly, double-dose regimens demonstrated marked increase of homologous virus reactivities (160 HI titers for both vaccines) while serum cross-reactivity was minimal (20–40 HI units) indicating poorly induced cross-immune responses despite booster immunizations (Fig. 2).

**Figure 3:**
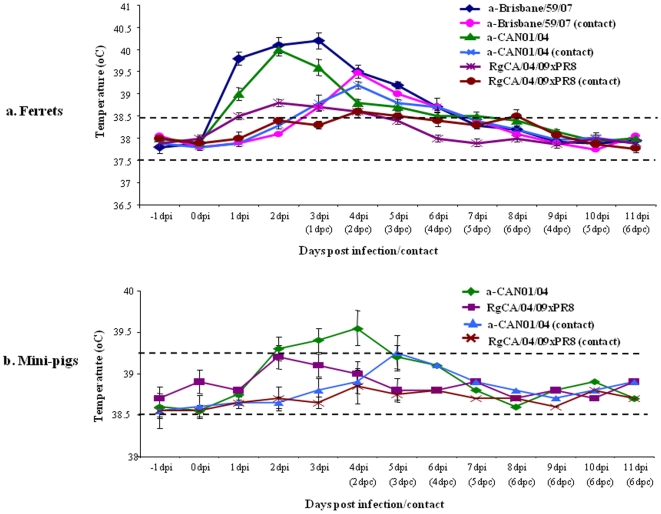
Monitoring of body temperature in ferrets and mini-pigs. Mean **b**ody temperatures of ferrets (a) and mini-pigs (b) infected with the pandemic (H1N1) 2009 virus (CA/04/09) or the recent Korean swine H1N1 (CAN01/04) isolate, including naïve contact animals, were monitored daily for 11 days post infection. The range of normal body temperatures are indicated as broken lines. Standard error bars are shown.

To ascertain whether such minimal levels of cross-reactive antibodies could provide any protection against infection with the pandemic (H1N1) 2009 virus, ferrets vaccinated twice with 7.5 µg/doses HA of a-Brisbane/59/07 or RgCA/04/09xPR8 were subjected to virus challenge experiments. Two-dose vaccinated hosts received CA/04/09 virus challenge at titers 10^5^ TCID_50_ two weeks after the last vaccination. Nasal wash specimens were collected from experimental animals on 2, 4, 5, 6, 7, and 8 dpi to examine the ability of the vaccines to hinder virus replication in the upper respiratory tract. To monitor transmission of the challenge virus from vaccinated to naïve animals through the air, two seronegative ferrets were added at 1 dpi in the same isolator constructed with perforated dividers preventing direct or indirect contact (5 centimeter distance) but could allow virus spread by aerosol transmission from infected ferrets. Elevated body temperatures were noted in a-Brisbane/59/07-immunized ferrets but not in RgCA/04/09xPR8 group from 1 to 4 dpi (39–40°C) (Fig. 3a) with some clinical signs of illness (i.e. runny nose and sneezing). Although the experimentally inoculated virus was detected in both vaccine groups starting at 2 dpi, higher virus titers were obtained from a-Brisbane/59/07-immunized ferrets compared to recipients of RgCA/04/09xPR8 (about 2 log_10_ EID_50_ difference) indicating unhindered active replication in the upper respiratory tract. The higher nasal titers from seasonal human vaccine recipients were also accompanied by prolonged virus shedding up to 5 dpi from initial virus detection and successful virus transmission at 2 days of post contact (dpc) (Table 3). Interestingly, aside from lowering nasal virus titers and reduced shedding, the RgCA/04/09xPR8-vaccinated group was also able to abrogate virus transmission to naïve contact ferrets. Consistent with the mice immunization, it appears that the seasonal human H1N1 could not completely protect vaccinated hosts (allowing virus replication and transmission) against infection with the pandemic (H1N1) 2009 virus.

**Table 3:** Nasal excretion of CA/04/09 in vaccinated ferrets.

Days	Viral titers (log_10_ EID_50_/ml)					
	a-Brisbane/59/07		a-CAN01/04		RgCA/04/09xPR8	
	Infected	Contact	Infected	Contact	Infected	Contact
−1	–	–	–	–	–	–
0	–	–	–	–	–	–
2	5 (0.3)*	–	4.5 (0.5)	–	3 (0.5)	–
4	3.5 (0.3	4 (0.5)	3 (0.3)	2.5 (0.3)	1.5 (0.3)	–
5	2 (0.5)	2.5 (0.3)	1 (0.3)	1.5 (0.3)	–	–
6	–	1 (0.5)	–	1(0.3)	–	–
7	–	–	–	–	–	–
8	–	–	–	–	–	–

Ferrets administered twice with 7.5 µl/dose HA of a-Brisbane/59/07, a-CAN01/04 or RgCA/04/09xPR8 were experimentally instillated i.n. with 10^5^ TCID_50_ CA/04/09 virus challenge in a 1.0 ml volume. Aerosol transmission of the test virus was monitored by adding seronegative contacts in an isolator with 5 cm perforated separation barrier. Nasal wash specimens were obtained at 2, 4, 5, 6, 7, and 8 dpi. Virus titrations were done in embryonated chicken eggs (log_10_ EID_50_/ml) where the limit of virus detection was set to 0.7 log_10_ EID_50_/ml. Dash marks indicate no virus detection.

* Standard deviation titers.

### Immunogenicity and Protectivity of a Swine H1N1 Vaccine (a-CAN01/04) in Mice and Ferrets against the Pandemic (H1N1) 2009 Virus

The current pandemic (H1N1) 2009 virus had been traced to have originated from pigs [9], [10]. Therefore, we tested whether an inactivated whole-virus swine H1N1 vaccine (a-CAN01/04, derived from a 2004 swine virus in Korea) could afford protection from infection among mice and ferrets. A/Swine/Korea/CAN01/2004 (CAN01/04) and CA/04/09 are both of swine-like origin but only share about 88% HA H1 sequence homology (Fig. 1). Following the same experimental procedure and set-up (vaccine regimens and HA protein content) as above, serum antibody responses in mice sera collected 2 weeks after the last vaccination with a-CAN01/04 were tested by HI assays. Robust serum HI antibody titers against the homologous vaccine virus were demonstrated by mice administered with two doses of immunization (as high as 320 HI units) (Table 1). However, when mice sera were tested against the CA/04/09 virus, two-dose vaccinations could only induce about 20–40 cross-reactive serum antibodies (against heterologous virus). After determining mean basal HI titers, mice were then experimentally inoculated i.n. with the pandemic (H1N1) 2009 virus. Viral titers in mice lung collected at 2, 4, 6, and 8 dpi indicated that a-CAN01/04 could not inhibit viral replication. However, relatively lower lung titers (about 1 to 2 log_10_ EID_50_ difference) were obtained compared to the mock-vaccinated group, a pattern similarly observed in seasonal human H1N1 vaccine recipients (Table 2).

Among a-CAN01/04 vaccinated ferrets, receipt of two doses (containing 7.5 µg/dose of HA, adjuvanted) elicited relatively high HI response (∼60 HI units) to the pandemic (H1N1) 2009 virus (Fig. 2). In contrast, homologous reactivity could be initially detected on single dose group (HI titer 30) which was robustly boosted in two-dose administrations (320 HI units). CA/04/09 virus challenge was also performed among two-dose vaccine groups. Clinical signs of illness were observed in challenged ferrets, such as inactivity and increase in body temperatures lasting for 2 days, were only noted in a-CAN01/04-vaccinated ferrets (Fig. 3a). Nasal washes collected at indicated time points demonstrated excretion of the challenge virus at peak titers of 4.5 log_10_ EID_50_ at 2 dpi persisting up to 5 dpi (Table 3). This substantially high and persistent nasal virus shedding could be accounted to the aerosol transmission of CA/04/09 into naïve animals at 2 dpc (at titers 2.5 log_10_ EID_50_).

### Immunogenicity and Protectivity of a-CAN01/04 and RgCA/04/09xPR8 in Miniature Pigs against the Pandemic (H1N1) 2009 Virus

**Figure 4:**
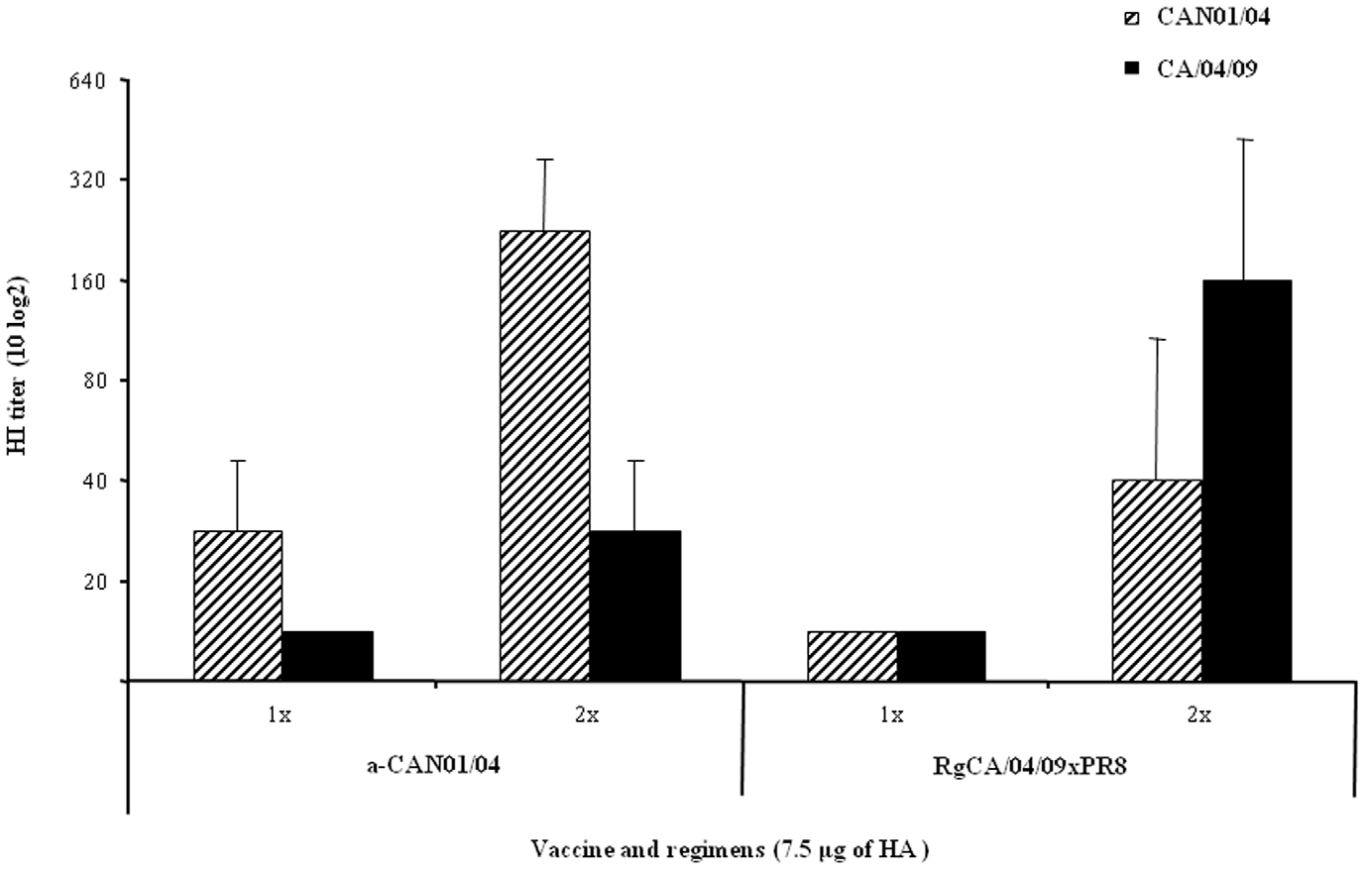
Hemagglutinin inhibition (HI) titers against the pandemic (H1N1) 2009 or Korean swine H1N1 virus in mini-pigs vaccinated with two doses of a-CAN01/04 or RgCA/04/09xPR8. Groups of two eight-week-old specific pathogen-free mini-pigs were vaccinated intramuscularly with one or two doses each of inactivated vaccines containing 7.5 µl/dose of HA with 2% of aluminum hydroxide adjuvant, administered 2 weeks apart. Sera were collected from recipients after 2 weeks the last vaccine was administered and mean antibody titers against CAN01/04 or CA/04/09 virus were determined by HI assays expressed as the reciprocal of the highest dilution of serum that inhibits 8 HA units of virus (e.g., as 80 versus 1∶80) with <20 HI units as the limit of detection. Data are mean ± standard deviation titers.

Pigs have demonstrated that they are susceptible to the pandemic (H1N1) 2009 virus under experimental settings [6], [11], [12] or through natural conditions [13]. Therefore, we also evaluated the vaccine efficacy of the recent swine vaccine strain (CAN01/04-like) in miniature pigs (mini-pigs). Groups of 2 specific-pathogen-free (SPF) mini-pigs were immunized once or twice with a-CAN01/04 or RgCA/04/09xPR8 containing 7.5 µg/dose of HA protein, in a 2-week interval for two-dose groups. Two weeks after their last vaccination, mean HI titers of each group against CA/04/09 or CAN01/04 were determined in swine sera. The induction of HI titers against a homologous or heterologous H1N1 virus was elevated in a dose-dependent manner. Single dose of a-CAN01/04 in mini-pigs elicited mean anti-HA titer of ∼30 against the CAN01/04 virus (homologous response) but the 2 dose schedule was more effective in raising serum antibodies (mean HI titer 240) (Fig. 4). When swine sera were tested against CA/04/09 (heterologous response), mean HI titers could not go beyond 40 units regardless of the regimen. In contrast, only double-dose administration with RgCA/04/09xPR8 effectively elevated serum antibody responses against CAN01/04 and CA/04/09 at mean titers 40 and 160, respectively (Fig. 4).

Subsequent to HI titration, all two-dose vaccinated animals received CA/04/09 virus challenge at titers 10^5^ TCID_50_/ml two weeks after the last vaccination. Nasal swab specimens were collected from experimental animals on 2, 4, 5, 6, 7, and 8 dpi to examine the ability of the vaccine to impede virus replication in the upper respiratory tract. Except for loss of appetite, no other remarkable sign of disease or changes in body temperature was observed in all vaccinated swine (Fig. 3b). Both of the vaccine groups started to shed the virus at 2 dpi but substantially higher titers (∼1 to 2 log_10_ EID_50_) were obtained from a-CAN01/04-vacccinated animals that lasted up to 5 dpi compared to the RgCA/04/09xPR8 group (Table 4). Transmissibility of the challenge virus from vaccinated animals was also assessed by co-housing the experimentally inoculated animals with seronegative pigs a day after infection. Due to a perforated barrier, transmission was only permitted by aerosol droplets. All naive contact pigs of the a-CAN01/04 vaccine group were positive for virus detection from day 3 through 5 pc indicating aerosol transmissions in mini-pigs (at peak titers 2 log_10_ EID_50_). In contrast, RgCA/04/09xPR8-vaccine group was able to suppress aerosol transmission of the CA/04/09 virus, consistent to data obtained in ferrets (Table 3). Reciprocal challenge of vaccinated hosts (done separately in groups of mini-pigs but using the same vaccine dose and frequency) using 10^5^ TCID_50_/ml of CAN01/04 as the test virus reversed the results. The a-CAN01/04 vaccine recipients blocked the transmission of the swine H1N1 virus (CAN01/04) which the RgCA/04/09xPR8 antigen failed to suppress (Table 4, panel 3 and 4).

**Table 4:** Nasal excretion of CA/04/09 and CAN01/04 in double-vaccinated mini-pigs.

Days	Viral titers (log_10_ EID_50_/ml)							
	CA/04/09 Challenge				CAN/01/Challenge			
	a-CAN01/04		RgCA/04/09xPR8		a-CAN01/04		RgCA/04/09xPR8	
	Infected	Contact	Infected	Contact	Infected	Contact	Infected	Contact
−1	–	–	–	–	–	–	–	–
0	–	–	–	–	–	–	–	–
2	5 (0.3)*	–	3 (0.3)	–	2 (0.3)	–	4 (0.5)	–
4	5 (0.5)	2 (0.3)	1.5 (0.5)	–	1 (0.2)	–	3.5 (0.2)	3 (0.3)
5	3 (0.3)	2 (0.3)	–	–	–	–	2 (0.3)	2.5 (0.3)
6	–	1 (0.3)	–	–	–	–	–	1 (0.2)
7	–	–	–	–	–	–	–	–
8	–	–	–	–	–	–	–	–

Two weeks after the last vaccination with a-CAN01/04 or RgCA/04/09xPR8 (7.5 µg/dose HA), 2-dose group mini-pigs received 1.0 ml 10^5^ TCID_50_ CA/04/09 virus challenge i.n. Nasal swabs were collected at 2, 4, 5, 6, 7, and 8 dpi. Transmission of the test virus through the air was also monitored in a similar set-up done in ferrets. Virus titers were calculated in embryonated chicken eggs (log_10_ EID_50_/ml) and the limit of virus detection was set to 0.7 log_10_ EID_50_/ml. Dash marks indicate no virus detection.

* Standard deviation titers.

**Figure 5:**
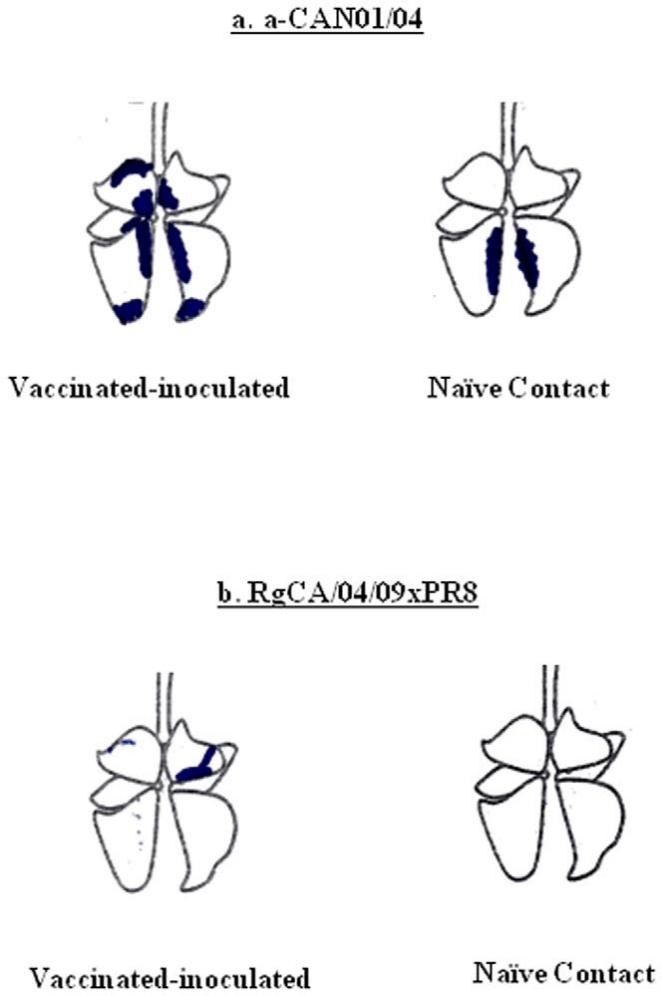
Gross pathological examination of lung tissue samples from infected and contact mini-pigs after challenge with the CA/04/09 virus. Lungs of 2-dose vaccinated [7.5 µg HA of either a-CAN01/04 (a) or RgCA/04/09xPR8 (b)] and subsequently challenged mini-pigs, including naïve contact hosts, were harvested at 5 dpi to examine gross tissue morphological features after infection with the pandemic (H1N1) 2009 virus.

To examine the pathological changes of each experimental animal after CA/04/09 challenge experiment, lungs were harvested (1 vaccinated and 1 contact) at 5 dpi. Gross lesions of a-CAN01/04-vaccinated mini-pigs already indicated apparent signs of regeneration. Both lungs are rather non-collapsed although there were diffused consolidation of cranial lobes and multifocal indications of bronchointerstitial pneumonia scattered through the accessory and caudal lobes (Fig. 5a). Minimal tissue consolidation could only be observed in the contact pig. Such overt lung pathological features were not observed in RgCA/04/09xCAN01/04 recipients including the naïve contact (Fig. 5b). Overall, these data suggest that similar to the seasonal human H1N1 vaccine, recent commercial swine H1N1 vaccine might not also offer protectivity against infection from the pandemic (H1N1) 2009 virus. For comparison, however, the a-CAN01/04 vaccine appears to be more immunogenic to the CA/04/09 virus than a-Brisbane/59/07.

## Discussion

Since the causative pandemic (H1N1) 2009 influenza virus is considered a novel strain, it appears that currently available human influenza virus vaccines could not elicit cross-reactive antibodies to the current pandemic virus [19], [20], [21]. Although receipt of adults with seasonal trivalent influenza vaccine generally resulted in a small increase in antibodies against the pandemic virus, it was not quite certain whether it is enough to provide any protection against infection [19]. We therefore evaluated the immunogenicity of a recent human seasonal H1N1 vaccine in mice and ferrets and formally investigated its cross-protective efficacy through wild-type virus challenge. On the other hand, since the involvement of animals, particularly pigs, to the epidemiology and spread of the virus is equally important, we also found it urgent to determine whether an inactivated and adjuvanted whole-virus prototype swine H1N1 vaccine would be efficient for veterinary use. For better evaluation and comparison, a pandemic H1N1 influenza vaccine generated by reverse genetics was also included (RgCA/04/09xPR8).

The Brisbane/59/07 virus is one of the recommended vaccine seed strain by the WHO against seasonal human H1N1 virus infections in the southern (2009 influenza season) and northern (2008–2009 and 2009–2010 seasons) hemispheres. On the other hand, the prototype swine H1N1 vaccine (a-CAN01/04) was prepared from a Korean swine virus isolated in 2004 [24]. The HA1 portion of the hemagglutinin molecule of CA/04/09 only share about 70% and 88.4% of amino acid sequence identities with Brisbane/59/07 and CAN01/04, respectively. Such low degree of genetic relatedness of the HA proteins could have contributed to the poor cross-reactivity of the two vaccines against the pandemic (H1N1) 2009 virus. Therefore, obtained results were not unexpected. Only two doses of a-CAN01/04 in mice were able to elicit detectable amount of cross-reactive antibodies (20–40 HI titers) compared to a-Brisbane/59/07 which could not induce any detectable HI titers beyond the detection limit regardless of the vaccine dose or frequency of administration (Table 1). At two-dose 7.5 µg HA, swine H1N1-vaccinated ferrets also demonstrated considerably higher cross-reactive antibody titers than seasonal human-vaccinated groups (60 versus 30 HI titers) (Fig. 2). Results obtained from a-Brisbane/59/07-vaccinated ferrets are consistent to the results obtained from previous antigenic testing in ferret post-infection antisera against currently circulating seasonal human A/H1N1 viruses [10] or in children and adult cohort subjects vaccinated with trivalent influenza vaccines [19], [20], [21].

However, intranasal virus challenge with the CA/04/09 virus in vaccinated animals indicated that the levels of cross-reactive antibodies detected by serologic assays were not sufficient to completely hinder active virus replication (Table 2 and 3). All recipients of both vaccines allowed virus persistence up to 8 dpi in mice lungs and continuous virus shedding through the nasal route until 5 dpi in ferrets. Furthermore, immunized ferrets could not abrogate the spread of the challenge virus via aerosol transmission into seronegative contacts. Consistently though, slightly lower nasal viral titers (about 0.5–1 log_10_ EID_50_) were obtained in swine H1N1-vaccinated mice and ferrets than in seasonal human H1N1-vaccinated hosts. When the a-CAN01/04 was further tested in mini-pigs, it can only induce limited cross-reactive immunogenicity against the CA/04/09 virus (Fig. 4) which could not suppress growth of the test virus in the upper respiratory tract and transmission to naïve contact host through the air (Table 4). Thus, the lack of cross-reactivity by serology could also be equated to a lack of cross-protective immunity among vaccinated hosts in our study. These also strongly suggest that neither the seasonal human nor the swine H1N1 vaccine could be effectively counter infection with the pandemic (H1N1) 2009 virus.

Of the three vaccines administered, it appears that only RgCA/04/09xPR8 effectively and consistently elicited high reactive serum antibody titers against the CA/04/09 virus in all the immunized hosts (mice, ferrets, and pigs) (Table 1, Fig. 2, and Fig. 3). When immunized sera were processed for micro-virus neutralization assays using 100 TCID_50_ of CA/04/09 in Madin-Darby canine kidney (MDCK) cells, administration of the reverse-genetics vaccine demonstrated efficient virus neutralizing activity which correlated comparably well with the vaccine-induced HI responses (data not shown). In contrast, neutralizing activities to the CA/04/09 virus was barely detected in either of the seasonal human or swine H1N1 vaccine recipients confirming poor cross-protection. However, this result is not so surprising due to identical antigenic match between the challenge and vaccine virus, but rather emphasizes the need for strain-specific vaccines. Sustained human-to-human transmission is a key requirement for pandemics and could result in the genesis of more pathogenic variants, as what happened with the 1918 pandemic virus (as reviewed in Reference 25). In response to live virus challenge, this important feature was considerably countered by the receipt of RgCA/04/09xPR8 in ferrets and pigs: virus growth and shedding in experimentally infected animals was limited which in turn reduced the chance of transmission of the test virus (Table 3 and 4). Collectively, these results indicate that the vaccine-induced antibody response detected was able to suppress the spread of infectious virions.

Receipt of either trivalent inactivated vaccine (TIV) or live attenuated influenza vaccine (LAIV) is the currently acceptable immunization strategies for the prevention and control of influenza infection. Although both types of vaccine are effective, the use of LAIV is considered more effective for its potential to induce broader and more durable protection against influenza (with regards to induction of influenza virus-specific serum and mucosal antibodies, cytotoxic T-cell and interferon responses) [26], [27]. Due to limited and unavailable resources, we were unable to evaluate the protective potential of seasonal influenza vaccines in live-attenuated form such that we cannot rule out the possibility of improved serologic cross-reactivity elicited by LAIV compared to our inactivated preparation. Hence, it will be interesting to investigate and compare in further studies with animal models the efficacy of inactivated and live-attenuated vaccines to provide protection against infection with the pandemic (H1N1) 2009 virus.

In summary, we report that contemporary human seasonal and veterinary H1N1 vaccines are unlikely to induce immunologic responses that could inhibit growth or transmission of the current pandemic (H1N1) 2009 virus. Undeterred efficient dissemination of the pandemic (H1N1) 2009 virus among humans could allow opportunities to acquire adaptive mutations producing more pathogenic variants. Alternatively, establishment in a new host could also facilitate the production of progeny viruses with deleterious consequences of unknown magnitude. Despite the increasing number of countries reporting animal infections, most notably among swine herds [13], no parallel studies evaluating the effects of seasonal vaccination on infection with the pandemic (H1N1) 2009 viruses in animal models (i.e., mice, pigs, ferrets) have been reported prior to this study. Given the importance of pigs as intermediate hosts for genetic reassortment [15] and their proven susceptibility to this strain, it is prudent that swine populations should also be protected to avoid their involvement in the epidemiology of the current pandemic virus. Thus, this study supports and warrants the development of strain-specific vaccines that will yield the optimal protection desired for humans and/or animals alike.

## Materials and Methods

### Ethics Statement

All experiments involving animal subjects were conducted in strict accordance and adherence to relevant national and international guidelines regarding animal handling as mandated by the Animal Use and Care by Laboratory Animal Research Center (LARC) in Chungbuk National University, a member of the International Animal Care and Usage Committee (IACUC), and in Bioleaders Corp.

### Viruses

The human pandemic H1N1 virus, CA/04/09, was obtained from St. Jude Research Hospital, USA. CAN01/04 (H1N1) is a recent swine influenza virus strain isolated from a Korean swine farm in 2004 [24] whose HA H1 gene is genetically related but phylogenetically distinct from CA/04/09 (Fig. 1). The Brisbane/59/07 vaccine seed virus was obtained from Green Cross, Korea. Viruses were 10-fold serially diluted and the 50% tissue culture infective doses (TCID_50_) were determined by infection in Madin-Darby canine kidney cells calculated by the method of Reed and Muench [28]. Stock viruses were kept at −80°C and thawed right before use. All experiments were conducted under approved biosafety level 3 (BSL-3+) facilities and conditions.

### Reverse Genetics and Vaccine Generation

The reverse genetics RNA transcription vector, pHW2000, and eight plasmids containing the cDNAs of influenza A/Puerto Rico/8/34 (H1N1) (PR8) were kindly provided by Dr. Robert G. Webster. The RgCA/04/09xPR8 reassortant virus containing the HA and NA genes CA/04/09 in the background of PR8 was generated by plasmid-based reverse genetics as described previously [29]. The rescued recombinant was confirmed by re-sequencing.

The CAN01/04, Brisbane/59/07, and RgCA/04/09xPR8 viruses were propagated in the allantoic fluid of a 10-day old embryonated chicken egg and purified by ultracentrifugation through a 25% and 70% sucrose cushion, at 30,000 X g in 4°C for 3 hours, as described previously [30]. Purified viruses were inactivated by treatment with 0.025% formalin in 4°C for at least one week which resulted in the complete loss of infectivity of the virus. Virus inactivation was confirmed by the absence of detectable infectious virus following inoculation of the vaccines into eggs. The quantity of HA protein in the vaccines were determined to be 30% of the total viral proteins by densitometric analysis of the viral protein bands separated by sodium dodecyl sulfate-polyacrylamide gel electrophoresis as described by Lu et al (1999) [31]. The human seasonal and swine H1N1 vaccines used in this study were designated as a-Brisbane/59/07 and a-CAN01/04, respectively.

### Vaccination and Virus Challenge

Four-week-old BALB/c mice were obtained from Samtaco (Seoul, Korea), eight-week-old specific-pathogen-free (SPF) outbred miniature pigs were from PWG Genetics Korea, Ltd (Pyongtaek, Korea), and 15- to 16-week-old ferrets were purchased from Marshall Bio Resources (New York, USA). All animals were seronegative for influenza A viruses by serologic assay. Mice (12 heads per group) were vaccinated intramuscularly with 1 or 2 doses of inactivated vaccines containing 1.77 or 3.5 µg/dose of HA containing 2% of aluminum hydroxide adjuvant in 200 ul volume, administered 2 weeks apart. Two weeks after the last immunization, mice were i.n. challenged with 30 µl 10^5^ TCID_50_ per milliliter of the CA/04/09 virus. Mini-pigs (n = 2 per group) and ferrets (n = 4 per group) were vaccinated intramuscularly with one or two doses each of inactivated vaccines that contain 7.5 µg/dose of HA with 2% of aluminum hydroxide adjuvant, administered 2 weeks apart. Intranasal instillation of 10^5^ TCID_50_ CA/04/09 virus challenge in a 1.0 ml volume (divided between two plastic syringes for separate inoculation of each nostril) was done after 2 weeks of receiving their last immunization. For transmission studies in ferrets and mini-pigs, animals (n = 2) were co-housed in adjacent transmission cages fitted in the same isolator (at a distance of 5 centimeters apart) that prevented direct or indirect animal contact but allowed influenza virus spread through aerosol contact from experimentally infected animals. All viruses and animal experiments including serologic testing were handled in a BSL 3+ containment facility approved by the Korea Centers for Disease Control and Prevention.

### Sera, Nasal Swab/Wash, and Tissue Collection

Sera from mice, mini-pigs, and ferrets were collected after 2 weeks of receiving their last vaccination, respectively, and stored at −82°C until use.

Lung tissue samples of mice were collected at 2, 4, 6, and 8 dpi and homogenized in 1X phosphate-buffered saline (PBS) containing antibiotics. Tissue homogenates were clarified by centrifugation at 12,000 g and supernatants were transferred to new tubes. Nasal washes (ferrets) and swabs (mini-pigs) were collected in 1X PBS with antibiotics after days 2 to 8 of virus challenge. All samples were immediately serially diluted 10-fold and then inoculated into 11-day-old embryonated chicken eggs for virus titration as computed by the Reed and Muench method with results expressed as log_10_ 50% egg infective dose per milliliter or gram of tissue collected (EID_50_/mL or EID_50_/g) [28]. The limit of virus detection was set to 0.7 log_10_ EID_50_/mL. Lungs of infected and contact pigs (one head per group) were harvested at 5 dpi for gross histopathological examination.

### Hemagglutination-Inhibition (HI) and Virus Micro-Neutralizing Assays

Hemagglutinin inhibition (HI) assays were done as described elsewhere [32]. Briefly, obtained sera were treated with receptor destroying enzyme (RDE) to inactivate non-specific inhibitors with a final serum dilution of 1∶10. RDE treated sera were serially diluted 2-fold and equal volume of virus (8 HA units/50 µl) was added to each well. The microplates were incubated at room temperature for 30 min followed by the addition of 0.5% turkey red blood cells. The plates were gently mixed and incubated at 37°C for 30 min. The HI titer was determined by the reciprocal of the last dilution that contained turkey RBCs with no agglutination. Virus neutralizing titers were determined by infection of MDCK cells and expressed as the reciprocal of the highest dilution of serum that gave 50% neutralization of 100 TCID_50_ of virus after incubation at 37°C for 72 h [33].
